# Grafting with Bone Substitute Materials in Therapy-Resistant Periapical Actinomycosis

**DOI:** 10.1155/2021/6619731

**Published:** 2021-02-25

**Authors:** Saeed Asgary, Leyla Roghanizadeh

**Affiliations:** Iranian Center for Endodontic Research, Research Institute of Dental Sciences, Shahid Beheshti University of Medical Sciences, Tehran, Iran

## Abstract

Actinomycosis can be one of the causes of persistent periradicular lesions. This is the report of a patient who was first referred with complaint of pain in maxillary right incisors. A standard root canal therapy was carried out. Unluckily, the patient returned with recurrent symptoms; therefore, surgical endodontic retreatment was decided. While the large periradicular lesion was curetted, a whitish yellow granule-like material came out from the periapical area that was submitted for histopathological examination. The apices of both maxillary right incisors were resected. Root-end cavities were sealed with calcium-enriched mixture (CEM) cement. Finally, the remaining large defect was filled with natural bone substitutes. Since the histopathological diagnosis revealed actinomycotic infection, oral penicillin V was prescribed for four weeks. At two-year recall, the bone healing process was completed. Apical actinomycosis can cause therapy-resistant lesions. Root-end surgery employing CEM and bone substitutes might be an effective method to help bone healing in large periradicular lesions.

## 1. Introduction

Actinomycosis is a slowly progressive bacterial infection. The causative bacteria include facultative anaerobic, filamentous, gram-positive bacilli that belong to the *Actinomyces* genus [1]. They can be present as the microbial flora of tonsillar crypts, gingival crevices, periodontal pockets, and dental plaques [1]. Cervicofacial actinomycosis usually establishes following a disruption of the mucosal barrier, dental manipulations, or trauma to the mouth, such as a tooth extraction or a mandibular bone fracture, although it may arise spontaneously in patients with poor dental hygiene [2, 3].

Bone defects can be created by several etiologic factors such as infections, tumors, or genetic disorders. Those lesions do not simply heal, and assistance of bone substitute materials for bone regeneration is usually essential [4]. The applied biomaterials/grafts include “(i) barrier membranes used for guided tissue regeneration (GTR), (ii) bone replacement grafts (such as allografts, xenografts, and alloplastic materials), and (iii) wound modifiers (such as enamel matrix derivative).” Combinations of these types have been applied too [5].

This report presents a case of a large persistent endodontically induced periapical defect around the root of a maxillary lateral incisor that has undergone root canal therapy (RCT), although no healing resulted from the primary treatment. Thus, a surgical endodontic retreatment was planned.

## 2. Case Presentation

**Figure 1:**
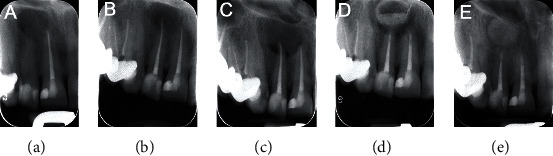
Periapical radiographs: (a) preoperative image of teeth #12, showing a large endodontic lesion; (b) immediate postendodontic radiograph; (c) at two-month follow-up, the patient returned with abscess formation and a larger periapical lesion; (d) applying bone substitute material during surgical retreatment; (e) at 6-month follow-up, bone healing can be observed.

A 50-year-old woman was referred to the endodontic department of a private dental clinic. She had pain and discomfort upon mastication associated with her upper right incisors as the chief complaint. Her medical history was noncontributory. Intraoral examination of the region showed that teeth #11 and #12 had composite resin restorations; tooth #12 was tender to percussion and palpation. The probing depths of both teeth were within normal limits (i.e., ≤3 mm), and no abnormal mobility was found. No swelling or sinus tract was detected in the buccal or lingual mucosa. Both teeth did not respond to sensibility tests, including an electrical pulp tester (Parkell, Edgewood, NY, USA), and cold test with Endo-Frost (Coltène-Whaledent, Langenau, Germany). In the radiographic evaluation (Figure 1(a)), tooth #11 had an adequate root canal filling and tooth #12 had a large well-defined radiolucent lesion.

Based on the clinical and radiographic findings, the diagnosis was asymptomatic apical periodontitis associated with a necrotic upper lateral incisor. Primary RCT was decided for tooth #12. The patient was informed about the diagnosis and the treatment plan. Informed consent was obtained.

On the treatment session, after local anesthesia with 2% lidocaine plus 1 : 80000 epinephrine (Darupakhsh, Tehran, Iran), access cavity was prepared, and treatment was continued with a rubber dam in place. There was no pus/exudate from the root canal. Cleaning and shaping by Flexofile (Dentsply, Maillefer, Switzerland) #15-40, accompanied by 5.25% sodium hypochlorite, were performed; then, the canal was obturated by a lateral condensation technique. Finally, the tooth was permanently restored with composite resin (Figure 1(b)).

**Figure 2:**
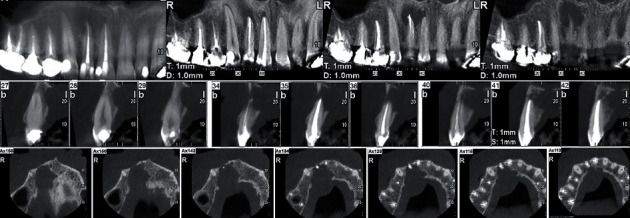
CBCT scan of the maxillary anterior region showing a large bony defect surrounding the periapex area of tooth #12, which extended from the distal aspect of the root of tooth #13 toward the mesial wall of the root of tooth #11.

After 2 months, the patient returned with no recovery from the signs/symptoms of tooth #12; clinically, a small localized abscess in the buccal vestibule and a large abscess in the palatal mucosa of the upper right incisors could be observed. Based on periapical radiograph (Figure 1(c)), with an adequate root canal filling, the size of the periapical lesion increased and the large bony defect extended toward the periapex of tooth #11. Cone-beam computed tomography (CBCT) was prescribed to scrutinize the associated region which showed a large radiolucent lesion in between the palatal aspect of teeth #13 and #11 (Figure 2). Considering all of the findings, the diagnosis was a therapy-resistant apical lesion, and surgical endodontic retreatment for both upper right incisors was judged to be favorable. The patient was thoroughly informed. She signed the informed consent form.

At the surgery appointment, the mouth was rinsed with 0.2% chlorhexidine and local anaesthesia via infiltration with 2% lidocaine plus 1 : 80000 epinephrine (Darupakhsh, Tehran, Iran) was administered. Then, a full mucoperiosteal flap was prepared and retracted. There was no buccal cortex over the root apex of tooth #11; after curettage of the granulation tissue, a through-and-through bony defect was appeared. During the procedure, a small whitish granule-like material was obtained. The specimen was stored in 10% formalin solution and submitted for histopathological examinations. Root-end resection/preparation for both incisors was carried out. Next, calcium-enriched mixture (CEM) cement (BioniqueDent, Tehran, Iran) was prepared according to the manufacturer's instructions and was delivered into the root-end cavities using a plastic instrument. After accomplishing the root-end fillings, the bony defect was filled with natural bovine bone grafting material (Cerabone, Botiss, Berlin, Germany). Following a confirmation radiography (Figure 1(d)), the flap was gently repositioned and sutured. Postsurgical recommendations were given to the patient.

**Figure 3:**
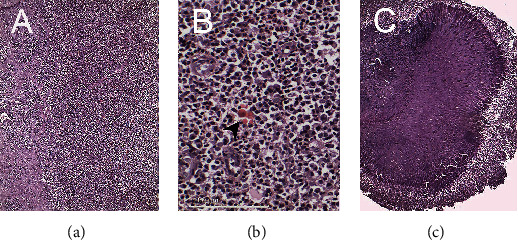
Pathological images: (a) granulation tissue (100× magnification); (b) showing in greater magnification (400×) collections of plasma cells, polymorphonuclear leukocytes, a few Russell bodies (which are marked with the arrow), and areas of erythrocyte extravasation; (c) club-shaped filaments in a radiating pattern demonstrating actinomycotic colonies (100× magnification).

Histopathological evaluations of sections of the specimen with conventional hematoxylin-eosin (H&E) staining (Figure 3) showed fragments of actinomycotic colony exhibiting club-shaped filaments arranged in a radiating rosette pattern with a basophilic central core and eosinophilic peripheral portion. In addition, sections show granulation tissue with large collections of plasma cells, polymorphonuclear leukocytes, areas of erythrocyte extravasation, and a few Russel bodies. The diagnosis was periapical granuloma with actinomycotic infection.

**Figure 4:**
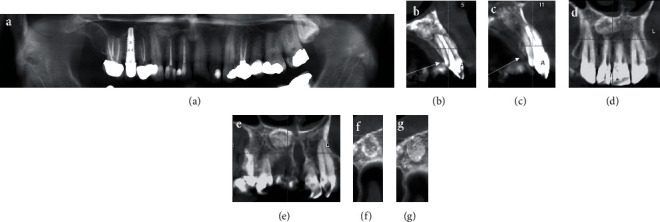
Two-year follow-up radiographic evaluation: (a) panoramic view: bone healing in the periapical areas of teeth #11 and #12; (b, c) axial views from teeth #11 and #12: bone augmentation in the palatal aspect of the maxilla; (d, e) frontal views of maxillary anterior teeth: there is no sign of radiolucent lesion around teeth #11 and #12 and bone substitute material can be observed; (f, g) transverse views of the area: successful bone augmentation.

After surgical intervention, the primary treatment outcome was satisfactory regarding symptom amelioration at the one-week follow-up. Based on the advice from her infectious disease specialist, the 500 mg tablets of penicillin V (one tablet per day for 4 weeks) was prescribed. At 6-month recall appointment, the tooth was in normal function, and there was no sensitivity to palpation or percussion. Radiographic examination (Figure 1(e)) demonstrated significant bone formation, and the periapical lesion was healed. After two years, the patient needed to take CBCT for implant surgery in posterior regions of both jaws. We made another study model for better assessment of periapical regions in right maxillary central and lateral incisors as two-year follow-up radiographic evaluation (Figure 4). The CBCT images show no radiolucent lesion in periapical areas of teeth #11 and #12. In addition, successful bone augmentation and healing can be observed (Figure 4).

## 3. Discussion

Actinomycosis in the oral cavity is an important disease to encounter. Sometimes, it is not timely diagnosed, due to general paucity of familiarity with the infection [1] and difficult culture of the causative bacteria, *Actinomyces* [6]. A precise and timely recognition of this infection requires a high degree of suspicion [1]. Furthermore, because of the resorptive potential of granulation tissue and extensive tissue destruction by actinomycotic infection, appropriate healing in apical actinomycosis is always demanding [1]. True diagnosis of this periapical infection can be reached only after surgical removal of the lesion and histopathological/microbiological examination. Definite diagnosis requires identification of the involved microorganisms such as *Actinomyces* species. However, commonly the infection has been diagnosed by the presence of sulfur granule, demonstrating actinomycotic colonies, obtained from endodontic surgery or through tooth extraction [7].

To treat and eliminate actinomycotic infection, combination of surgical removal of the involved tissue and suitable antibiotic therapy is necessary [6]. Penicillin is the traditional antibiotic of choice. The duration of antibiotic therapy can range from 4 weeks to 1 year based on the severity of the disease. Surgical management without antibiotic therapy might be associated with recurrence [1]. Recently, the concept that extraradicular infection may be established in the form of actinomycotic-like colonies and can cause treatment failure as an independent entity has been supported [8]. Endodontic surgery is an appropriate treatment plan for definitive removal of persistent extraradicular infections [9].

To decide eligibility for being a satisfactory root-end filling material, biocompatibility and sealing ability are crucial qualities. One of the biomaterials which has shown favorable characteristics in terms of sealability is CEM cement [10]. CEM not only can establish an effective bioseal but also has been able to stimulate osteogenesis and cementogenesis associated with regenerative periapical tissue responses [11]. CEM has high biostimulation potential for reproduction of dental hard tissues when it has been placed adjacent to live pulpal structure [12].

Another issue to discuss is that large bony defect secondary to endodontic surgeries may compromise the tooth function postoperatively. By application of GTR and/or bone graft approaches, the auxiliary materials can promote normal trabecular bone formation and also hinder migration of the proliferating oral epithelium into such lesions [13]. These techniques can improve the predictability of clinical, radiographic, and histological outcomes [14]. In this reported case, using bone substitute material resulted in successful healing. There are many studies in the literature in which large periradicular lesions with endodontic origin have been cured by the technique of GTR and bone grafting [13–15]. However, in some studies, just bone graft was applied and no membrane was used [16–18]. The outcome of endodontic surgeries in large lesions, especially in through-and-through defects, was more efficiently improved by the GTR technique [13]. In through-and-through defects, by this grafting approach, it would not be necessary to raise a flap on both sides of the alveolar process and would be technically easier to do than the application of membranes [18]. Finally, there is a need for more large-scale prospective clinical trials to assess added benefits of regenerative techniques for the outcomes of endodontic surgeries [15].

## 4. Conclusion

A therapy-resistant periapical lesion can be the result of an actinomycotic infection, which needs surgical intervention to promote the healing process. Using bone substitute material might facilitate healing and bone formation in large bone defects.

## Data Availability

The authors alone are responsible for the content and writing of the paper.
